# Quantitative Trait Locus Mapping and Identification of Candidate Genes Controlling Bolting in Spinach (*Spinacia oleracea* L.)

**DOI:** 10.3389/fpls.2022.850810

**Published:** 2022-03-30

**Authors:** Qing Meng, Zhiyuan Liu, Chunda Feng, Helong Zhang, Zhaosheng Xu, Xiaowu Wang, Jian Wu, Hongbing She, Wei Qian

**Affiliations:** ^1^Institute of Vegetables and Flowers, Chinese Academy of Agricultural Sciences, Beijing, China; ^2^Ilera Healthcare LLC, Waterfall, PA, United States

**Keywords:** spinach, bolting time, quantitative trait locus (QTL), QTL mapping, candidate gene

## Abstract

Spinach is a typical light-sensitive plant. Long days can induce early bolting, thereby influencing the regional adaptation, quality, and vegetative yield of spinach. However, the genes and genetic mechanisms underlying this trait in spinach remain unclear. In this study, a major quantitative trait locus (QTL) *q*BT1.1, was mapped on chromosome 1 using a BC_1_ population (BC_1a_) derived from 12S3 (late-bolting recurrent lines) and 12S4 (early bolting lines) with specific-locus amplified fragment (SLAF) markers and Kompetitive Allele Specific PCR (KASP) markers. The *q*BT1.1 locus was further confirmed and narrowed down to 0.56 Mb by using a large BC_1_ (BC_1b_) population and an F_2_ population using the above KASP markers and the other 20 KASP markers. Within this region, two putative genes, namely, *SpFLC* and *SpCOL*14, were of interest due to their relationship with flower regulatory pathways. For *SpCOL*14, we found multiple variations in the promoter, and the expression pattern was consistent with bolting stages. *SpCOL*14 was therefore assumed to the best candidate gene for bolting. Overall, our results provide a basis for understanding the molecular mechanisms of bolting in spinach and contribute to the breeding of diverse spinach germplasms for adaptation to different regions.

## Introduction

Spinach (*Spinacia oleracea* L.) is a diploid plant (2n = 2x = 12) of the Amaranthaceae family (Morelock and Correll, 2008). It was domesticated in Iran around 2,000 years ago (Rubatzky and Yamaguchi, 1997) and was first mentioned as the “herb of Persia” in China approximately 600 A.D. (Kuwahara et al., 2014). Spinach is an important and nutritious green leafy vegetable that is rich in carotenoids, folate, vitamin C, calcium, and iron (Lester et al., 2013). Spinach is also a good source of antioxidants and has one of the highest ORAC (oxygen radical absorbance capacity) values of any vegetable (Koh et al., 2012). It is typically consumed as a fresh, cooked or canned vegetable (Morelock and Correll, 2008; Ma et al., 2016). The reproductive process usually begins with bolting (the elongation of the stem) (Mutasa-Göttgens et al., 2010), which leads to decreased yields and low quality (Abe et al., 2014). Spinach is easily influenced by the photoperiod (Chun et al., 2000b), and bolts in spring (Tang et al., 2018). New slow-bolting spinach cultivars available to that can adapt to a wide range of photoperiods and climatic conditions (Bhattarai and Shi, 2021). Selecting the appropriate cultivars will improve the efficiency of breeding and production in spinach (Goreta and Leskovar, 2006).

Bolting refers to the rapid lengthening of the plant stem and is due to the coordinated effects of developmental and environmental factors (Chen et al., 2019). As a complex quantitative trait, bolting shows continuous phenotypic variation in many crops (Melchinger, 1998). Bolting is a transitional stage between vegetative growth and reproductive growth, and thus evaluating the genetics of bolting is essential for elucidating this phenomenon. Many key bolting and flowering genes have been identified and functionally characterized in *Arabidopsis*. *Arabidopsis* is characterized by inflorescence axis elongation-type bolting (Chen et al., 2019), which provides a reference for the study of bolting and flowering genes in spinach. Genetic studies in *Arabidopsis* have revealed that the genes controlling bolting and flowering are involved in, and can be assigned to, distinct regulatory pathways, including photoperiod, vernalization, gibberellin, autonomous, ambient temperature, and age (Fornara et al., 2010). These pathways are associated with plant developmental and environmental cues, such as photoperiod and temperature (Cho et al., 2017). One of the key genes affecting bolting and flowering is *FLOWERING LOCUS* C (*FLC*), which represses bolting and flowering by encoding the MADS-box protein in the vernalization pathway and *FLC* is expressed widely in the shoot apical meristem and leaves (Sheldon et al., 1999, 2000). The other key genes affecting bolting and flowering include *CONSTANS* (*CO*), which is involved in the photoperiod pathway. *CO* is the key gene accelerating bolting and flowering during long days (Suarez-Lopez et al., 2001), which acts upstream of *FLOWERING LOCUS* T (FT) in the photoperiod pathway (Dally et al., 2014). *CO* belongs to *CONSTANS-LIKE* (*COL*) proteins, called B-box (BBX) proteins (Griffiths et al., 2003). *COL*s are a class of zinc finger transcription factors that consist of a *CO*, *COL*, and TIMING OF CAB1 (CCT) domain (Abe et al., 2014). One *COL* (*SoCOL*1) and two *FLOWERING LOCUS* T (*FT*) homologs were isolated and characterized in the photoperiodic regulation of spinach (Abe et al., 2014).

It has been reported that flowering and bolting traits in spinach are greatly affected by long-day photoperiods and gibberellin (Zeevaart, 1971; Wu et al., 1996; Kim et al., 2000). Thus far, a few molecular markers and genes related to bolting and flowering in spinach have been reported. Chitwood et al. (2016) used 288 United States Department of Agriculture (USDA) spinach accessions as the association panel in this research and found three single nucleotide polymorphism (SNP) markers associated with bolting through genotyping-by-sequencing (GBS) technology and genome wide association study (GWAS). A draft genome sequence of spinach has been reported, and two quantitative trait loci (QTLs) associated with bolting have been obtained in the region from 44.7 to 50.5 Mb of chromosome 2 (Xu et al., 2017). Bhattarai et al. (2020) identified SNP sites associated with bolting and flowering on chromosomes 2, 3, and 5 by GWAS techniques with 300 USDA spinach accessions. Recently, a new spinach genome SOL_r1.1 have revealed three QTLs connected with bolting by double-digest restriction-site-associated DNA sequencing (ddRAD-seq) (Hideki et al., 2021). GWAS analyses of bolting and flowering traits yielded several associated regions across the six chromosomes and detected a region harboring genes encoding MADS-box transcription factors (SOV6g023690 and SOV4g008150) by the Monoe-Viroflay spinach genome (Cai et al., 2021). With the transcriptome sequencing of spinach bolting (Abolghasemi et al., 2021), more genes will be detected in the future research. These results suggest that spinach bolting is controlled by multiple QTLs or genes. However, details on the genetic mechanisms of bolting and flowering remained unclear in spinach, and no reliable molecular markers have been developed for the molecular marker-assisted selection (MAS) of slow bolting traits in spinach breeding.

Quantitative trait locus mapping is a powerful approach to dissect the genetic architecture of complex traits (Mauricio, 2001), and has been used to identify potential genes by revealing the relationship between the genotype (based on molecular markers) and phenotype (Salvi and Tuberosa, 2005). In spinach, QTL mapping has largely been used to investigate in: sex-determining locus (Khattak et al., 2006), nitrogen use efficiency (Chan-Navarrete et al., 2016), leaf color (Cai et al., 2018), fruit spines (Liu et al., 2021a), and leaf-related traits (Liu et al., 2021b). Moreover, the bolting trait has been reported in many crops by QTL mapping, such as *Brassica napus* L. (Fu et al., 2020; Xu et al., 2021), *Beta vulgaris* (Tränkner et al., 2017), wheat (Buerstmayr et al., 2009) and so forth. In our previous study, the early bolting inbred line 12S4 and the late-bolting line 12S3 were used as parents to develop segregated populations, and a high-density spinach genetic linkage map with 4080 specific-locus amplified fragment (SLAF) markers (Qian et al., 2017) was constructed using a derived BC_1a_ population (*N* = 148). The objectives of the current study were to map the bolting gene through SLAF-based and KASP-based QTL mapping approaches and identify the candidate genes controlling the bolting trait using the BC_1b_ and F_2_ populations. This study will be help elucidate the genetic mechanisms of bolting, which may lay the foundation in MAS bolting behavior in spinach breeding.

## Materials and Methods

### Plant Material and Phenotyping Evaluation of Bolting Time

Two inbred lines, 12S3 and 12S4, which exhibit significant differences in bolting, were selected as the parents. Line 12S3, with extreme resistance to bolting, was used as the female and recurrent parent, while the early bolting line 12S4 was used as the male parent to develop a BC_1_ and an F_2_ population (Qian et al., 2017). The two parental lines, the derived F_1_ line, and the 148 BC_1_ individuals (BC_1a_) were planted in a field in spring 2015 for primary mapping. In addition, 200 BC_1_ progenies (BC_1b_) and 150 F_2_ progenies were planted in the same location in spring 2020 in natural conditions for validation of QTLs and narrowing down of the QTL regions. All of these materials were developed and tested by the Spinach Research Group, Institute of Vegetables and Flowers (IVF), and Chinese Academy of Agricultural Sciences (CAAS).

Each individual plant was visually inspected daily, and the bolting date was determined as the date that a stem of a plant was seen to be at least 5 cm in length (Fu et al., 2020). The bolting time (BOT) was then determined as the period from the sowing date to the bolting date. The phenotypic data of all plant materials in these experiments were analyzed with Excel 2013 (Microsoft Corp., Redmond, United States) for calculating the mean, standard error (SE), and coefficient of variation (CV) in each line or population.

### DNA Extraction

At the four true-leaf stage, fresh young leaves were collected from each plant of the F_1_, BC_1a_, BC_1b_, F_2_ populations, and parents, immediately frozen in liquid nitrogen, and stored in a –80°C freezer. Genomic DNA was extracted from each plant using the cetyltrimethyl ammonium bromide (CTAB) method (Murray and Thompson, 1980). The DNA concentration and quality were assessed using a ND-2000 spectrophotometer (Thermo Fisher Scientific, Wilmington, DE, United States) and 1.0% agarose gel electrophoresis, respectively.

### Specific-Locus Amplified Fragment Library Construction for High-Throughput Sequencing

Specific length amplified fragment sequencing (SLAF-seq) is an efficient method of large-scale genotyping developed on the basis of high-throughput sequencing technology and reduced representation library (RRL). In brief, an SLAF pilot-experiment was first designed to improve the efficiency of SLAF-seq, which considered the uniform distribution and avoided the duplication of SLAFs. Next, according to the pre-experiment, the SLAF library was conducted as follows The genomic DNA from each sample was completely digested by the two restriction enzymes - *Rsa*I and *Hae*III (New England Biolabs, NEB). After digestion, the DNA fragments were repaired with adenine and duplex tag-labeled sequencing adapters. Twenty polymerase chain reaction (PCR) cycles were used to enrich the concentration of fragments and the PCR products were then purified and pooled. The sample was performed by 2% agarose gel electrophresis (120 V, 60 min). After gel purifcation, DNA fragments of 364–414 bp were excised and diluted for paired-end sequencing. Finally, the selected SLAFs were sequenced on an Illumina High-seq 2500 sequencing platform (Illumina, Inc.; San Diego, CA, United States).

The analysis of SLAF-markers followed the procedures described by Sun et al. (2013). All SLAF paired-end reads were clustered on the basis of sequence similarity, which was detected by BLAST (-tileSize = 10, -stepSize = 5). Sequences with over 95% identity were grouped in one SLAF locus. SLAFs with two to four tags were deemed as polymorphic SLAFs.

### Single Nucleotide Polymorphism Molecular Marker Analysis and Genotyping

The SNP molecular markers were obtained from 4080 SLAF markers from the spinach high-density genetic map constructed by Qian et al. (2017), following which a total of 300 KASP primers was designed by the LGC company (Shanghai, China), and the slow bolting parent 12S3 and early bolting parent 12S4 were tested (Liu et al., 2021b). A subset of KASP primers were selected and used to genotype the BC_1a_.

For the KASP assays, each sample contained 2.5 μL 2 × KASP Master mix, 0.07 μL KASP Assay mix, and 2.5 μL genomic DNA diluted to 20–30 ng/μL. The reaction system was as follows: 94°C for 15 min, 10 cycles of 94°C for 20 s and 61°C (0.6°C drop per cycle) for 60 s and a further 26 cycles of 94°C for 20 s and 55°C for 60 s. An additional three cycles of 20 s at 94°C and 60 s at 55°C were executed if the results of the initial KASP thermal cycles did not acquire sufficiently defined genotype clusters. In addition to DNA samples, two no-template controls (NTCs) were included on each 384-well PCR plate. All plates were read below 40°C in a 7900 HT Fast Real-Time PCR System (Applied Biosystems), and the data were analyzed using SDS2.3 software (supplied by Applied Biosystems) (Semagn et al., 2014).

### Linkage Map Construction and Quantitative Trait Locus Mapping

The SNP markers were selected with no segregation distortion, and markers with more than 25% missing data were also excluded. The valid markers were then used to construct the linkage map from the BC_1a_ population using JoinMap 4.0 software (Van Ooijen, 2006). All markers were firstly grouped based on a threshold of LOD = 3.0, while all other settings were left at their default values.

The BC_1b_ and F_2_ populations were used to confirm and narrow down the predicted region, and other KASP markers were developed based on the SNP variation between the two parents around the initial QTL area. The QTLs for bolting were also detected using QTL IciMapping 4.2 software (Meng et al., 2015) based on the phenotype of 148 BC_1a_ individuals. The Composite Interval Mapping of ADDitive QTL (ICIM-ADD) method was used for QTLs. The parameters were as follows: a step in 1 cM, probability in stepwise regression of 0.001, and LOD = 3.0. The final QTLs were named based on the method of McCouch et al. (1997): “ *q*” + the English abbreviation of the trait + the chromosome number + “ . “ the QTL number.

### Candidate Gene Analysis and Real-Time Polymerase Chain Reaction of Bolting Time

Based on the of spinach genome annotations (version Sp75) in SpinachBase,^1^ the genes related to bolting and flowering within the identified interval were selected for further analysis. The full-length RNA was extracted at the 12-leaf-stage and the promoters of the candidate genes were sequenced between the two parents. The specific primers were designed by Primer3 plus^2^ (Table 2). The candidate genes were cloned and the sequences were aligned by MUSCLE software.^3^ Finally, the gene structure was elucidated based on the re-sequenced result.^4^

Quantitative real-time PCR was employed to evaluate the expression of the candidate genes from the seedling to bolting stages. Leaf tissue of the 12S3 and 12S4 lines was collected at 6, 9, 12, 15, and 18 weeks until both parents began bolting in spring of 2021 (12S3 bolted 18 weeks after planting; 12S4 bolted 15 weeks after planting) (Figure 7B), and the total RNA was extracted using a Plant Total RNA Mini Kit (GeneBetter Biotech, Beijing, China^5^). The cDNA was synthesized from 500 ng total RNA with a TranScript One-Step gDNA Removal and cDNA Synthesis Kit (TransGen Biotech, Beijing, China^6^). Three independent biological and three technical replicates of each period were performed and analyzed. The synthesized cDNA was subjected to quantitative real-time (qRT)-PCR analysis using a QuantStudio™ 12 K Flex Real-Time PCR System (Applied Biosystems) with SYBR Fast qPCR Mix (TaKaRa^7^). The reaction mixture contained 70 ng template cDNA, 0.2 μM of gene-specific primer (Table 2), 0.2 μM ROX Reference Dye II, 3.4 μL ddH_2_O, and 5 μL 2 × SYBR Fast qPCR Mix in a 20 μL volume. The qRT-PCR was performed at 95°C for 30 s, followed by 35 cycles of 95°C for 15 s and 60°C for 1 min. The relative expression was calculated using the 2^–ΔΔCT^ method. *SpActin* was used as the reference gene (Lee and Zeevaart, 2002).

## Results

### Bolting Time Analysis and Mapping of Quantitative Trait Loci Controlling Spinach Bolting

**FIGURE 1:**
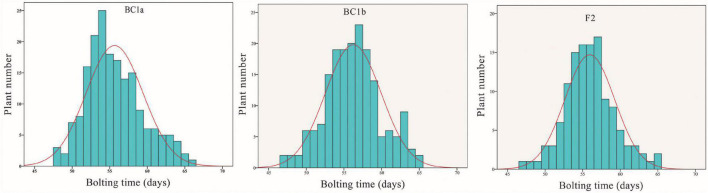
The distribution of days to bolting in BC_1a_, BC_1b_, and F_2_ spinach plants.

In 2015, the bolting time of line 12S3 and 12S4 was on average 62 (60–65) and 46.5 (45–48) days, respectively, indicating differences in the bolting time of the parents. From the BC_1a_ line, the bolting time ranged from 48 to 66 days, with an average of 54.5 days. The bolting time of 155 BC_1b_ and the 123 F_2_ individuals was from 47 to 65 days, with a mean value of 57.6 days in BC_1b_ and 55.8 days in the F_2_ populations. The phenotypic traits are summarized in Table 1 and Supplementary Table 1. Moreover, these segregating populations showed continuous variation in bolting time, suggesting that the bolting trait has a quantitatively inherited character in spinach (Figure 1 and Supplementary Table 2).

**TABLE 1:** QTL analysis of spinach bolting.

QTL	Strategies		Closest marker	Position (cM)	Marker interval	LOD	PVE (%)	Add
*q*BT1.1	SLAF-seq	BC_1a_	Marker2552708	282	Marker2552708 – Marker1611427	16.3902	49.0697	0.4744
*q*BT1.2	KASP	BC_1a_	KM3677664	105	KM3677664 – KM41831444	3.4608	8.8993	0.1976
*q*BT1.1			KM3309304	166	KM3309304 – KM3363916	13.3856	40.8646	0.4236
*q*BT1.1	KASP	BC_1b_	KM3309304	31.5384	KM3309304 – KM3363916	10.5623	41.9909	–4.4104
*q*BT1.1	KASP	F_2_	KM3309304	33.1346	KM3309304 – KM3363916	19.9202	51.1984	3.7826

**TABLE 2:** The primers used to screen candidate genes controlling spinach bolting trait.

Name	Sequencing
04942-m-1F	CCTTCCCGGACACAACTTGA
04942-m-1R	AACGTTCCCAATGCTTTGCC
04967-m-1F	CCTTTTCCACAAACCCATCCT
04967-m-1R	GCTAGCTAGCTAATACATGGCTG
04942-D-5F	TGGTACATATAGGCGCCACG
04942-D-5R	GTAAAAGAGAGCGGGGGTCG
04967-D-3F	TATTGGGTCGGGTTCGCTTC
04967-D-3R	AAAGCTTAGCGGTGTCAGCT
04967dIN1-1F	CCATAGGGGTAAATTGAAATTGAAGA
04967dIN1-1R	ACCAACCTACACCAAGAAGTT
04942-q3F	TAGTCCCACCAATCCTCCTATAC
04942-q3R	CTTCACTTTCACGGTACCCAATA
04967-q5F	ACCGGAGAACAACAATGTGG
04967-q5R	ATGTCGGCCTCTGTTCTTACTC
SpActin-F	GGTGATGGTGTTAGTCACAC
SpActin-R	AATGATGGCTGGAAGAGAAC

**FIGURE 2:**
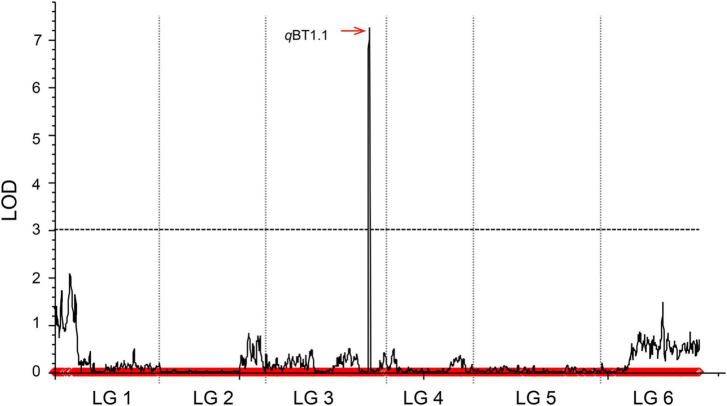
Mapping QTLs controlling spinach bolting trait using a high-density genetic linkage map constructed with SLAF markers.

In our previous study (Qian et al., 2017), a total of 4080 SLAF markers for 148 BC_1a_ individuals were acquired by SLAF-seq, and the linkage groups were coded with six linkage groups (P01–P06) in a total length of 1125.97 cM, which matches the spinach chromosome numbers (Supplementary Table 3). After the exclusion of missing and disqualified data, 130 BC_1a_ individuals were finally used to map the QTLs (Supplementary Table 3). Combining the SLAF high-density genetic map with bolting time in 130 BC_1a_ progenies, a major QTL (named *q*BT1.1), which contributed 49.07% of the phenotypic variance (PVE) (Table 1), was identified at the interval 15.82–18.97 cM on LG3 between two adjacent SLAF markers (Marker 2552708 and Marker 1611427), with an LOD score of 16.39. Based on the spinach genome Sp75 (Xu et al., 2017), this QTL was mapped on chromosome 1 in the region of 47.72–50.61 Mb (Figure 2).

**FIGURE 3:**
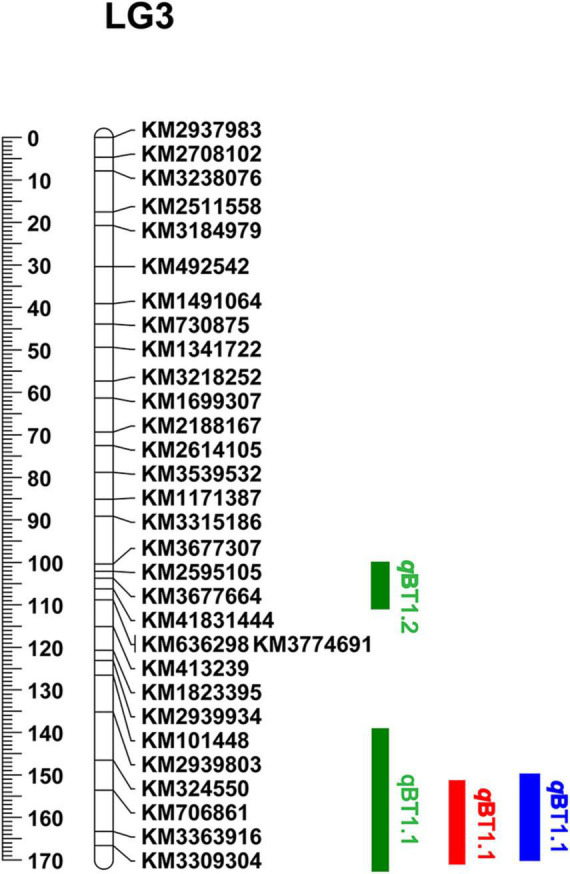
QTL results of bolting using KASP genetic linkage map. The color Green, Red, and Blue respect BC_1a_, BC_1b_, and F_2_ populations, respectively.

A total of 181 informative SNP markers (Liu et al., 2021b) and 147 BC_1a_ individuals were selected for KASP-based linkage analysis (Supplementary Table 4). Based on the 181 KASP markers and screened 127 BC_1a_ plants, two QTLs (*q*BT1.2 and *q*BT1.1) were mapped to 103.5–105.5 cM and 163.5–166.0 cM on LG3 (Figure 3) and were located at 41.44–42.02 Mb and 46.76–49.12 Mb on chromosome 1, respectively. The LOD scores were 3.46 and 13.39, explaining 8.90 and 40.86% PVE, separately (Table 1). In 2020, the same 181 KASP markers were used in 185 BC_1b_ and 112 F_2_ populations and they were co-located in the same area between KM706861 and KM3309304 (Figure 3). The LOD score was 10.5623 and explained 41.99% PVE in BC_1b_ individuals while the figures were 19.9202 and 51.20% in F_2_ plants, respectively (Table 1).

### Fine-Mapping of Spinach Bolting

The SLAF-based and KASP-based QTLs in the two strategies indicated that *q*BT1.1 was a stable locus that could be used for fine-mapping and cloning. The SNP variations were explored in the sequences at this region of 12S3 and 12S4. The raw reads were first-filtered by fastp 0.12.0 (Chen et al., 2018) and the alignment data were obtained on the spinach genome Sp75 (Xu et al., 2017) by BWA 0.7.17-r1188 (Li and Durbin, 2009). The vcf files were finally generated by Samtools/Bcftools 0.1.19 – 44428 cd (Li et al., 2009). To further refine the mapping region, the KASP markers were developed from 40 to 51 Mb on chromosome 1 by the file.

**FIGURE 4:**
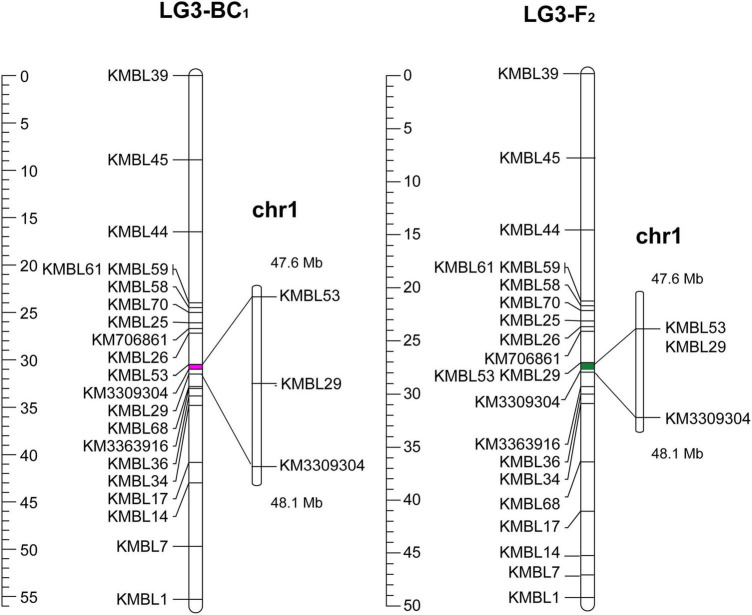
QTL mapping of BC_1b_ and F_2_ population: the left was the fine mapping of BC_1b_ population; the right was the fine mapping of F_2_ population.

After eliminating the invalid segregation data, 20 efficient KASP markers were designed for fine mapping (Supplementary Table 5). In the expanded BC_1_ population (BC_1b_), 185 individuals were obtained in 2020, and then 16 recombinant plants were ultimately acquired by the KASP genotyping. A major QTL (*q*BT1.1) for bolting time was verified between KMBL53 (31.0 cM) and KM3309304 (31.5 cM) (Figure 4). Furthermore, in the F_2_ population in 2020, the 112 plants were used to map the QTLs for bolting time, and nine recombinant individuals were obtained. A major QTL in the F_2_ populations was also fine-mapped in the interval of KMBL53 (32.4 cM) and KM3309304 (33.1 cM). In conclusion, a QTL named *q*BT1.1 was detected with a 0.56-Mb region between KMBL53 (47.56 Mb) and KM3309304 (48.12 Mb) on chromosome 1 (Figure 4).

### Screening for Candidate Genes Controlling Spinach Bolting Trait

**FIGURE 5:**
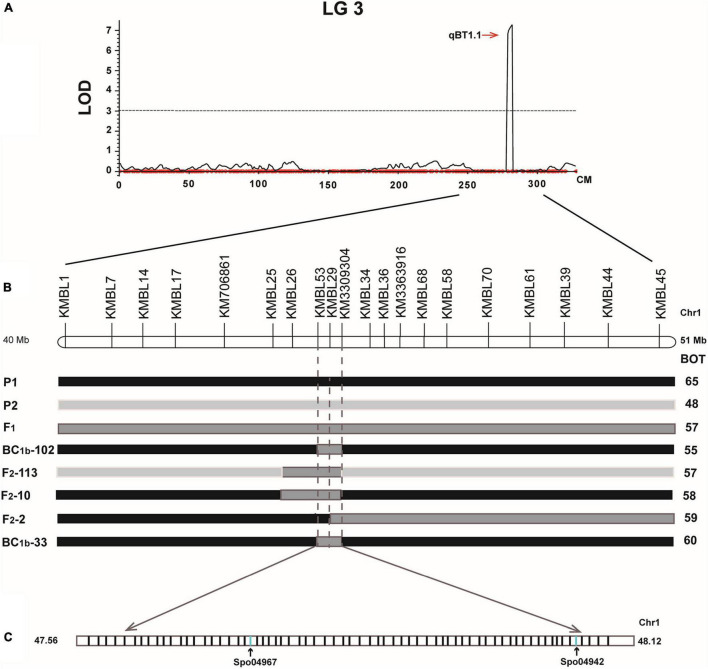
Fine mapping QTLs controlling spinach bolting trait. **(A)** One QTL was mapped in LG3 using the BC_1a_ population; **(B)** fine mapping of the QTL controlling spinach bolting trait using the BC_1b_ and F_2_ populations; **(C)** two candidate gene were identified in the interval from 47.56 Mb and 48.12 Mb of spinach chromosome 1.

A total of 68 genes were located in a 560 kb region based on the spinach genome (version Sp75)^8^ (Supplementary Figure 1 and Supplementary Table 6). Among these genes, two genes were unannotated, 23 genes encoded various enzymes, and four genes had transmembrane structure. Two genes *Spo04942* and *Spo04967* were found to be homologs of bolting and flowering genes in *Arabidopsis* (Table 2, Figure 5, and Supplementary Table 6), and thus could potentially be the candidate genes controlling the spinach bolting trait.

**FIGURE 6:**
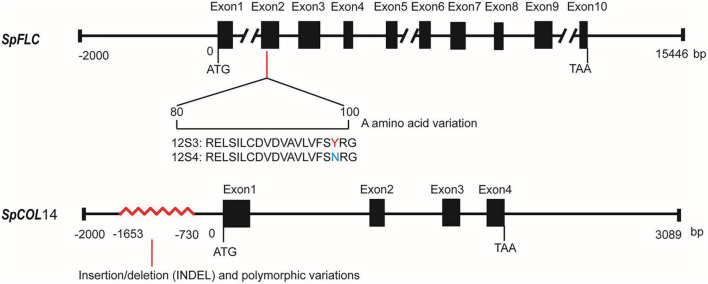
A schematic of the variations in *SpFLC* and *SpCOL*14 between the two parents.

*Spo04942* is MADS-box transcription factor that is homologous to *Arabidopsis FLC* and the sugar beet *FLC* homolog *FLC*-*LIKE* 1, thus was renamed as *SpFLC*. In the coding area, there were two synonymous SNP variations and one non-synonymous SNP variation that led to the change from tyrosine (12S3) to asparagine (12S4) at position 98 in the domain area (Figure 6). In the 2-kb upstream non-coding sequences, no difference was found between the two parents. The gene *Spo04967* may encode a zinc finger protein similar to *CONSTANS-LIKE* 14, which belonged to the *COL* family, and thus was named as *SpCOL*14. With Sanger sequencing, no variation was found in the coding region of *SpCOL*14 between 12S3 and 12S4. However, from the 2-kb upstream non-coding region, variations of about 900 bp were found between 12S3 and 12S4 (–730 bp to –1653 bp) (Figure 6) that may affect the expression of *SpCOL*14.

**FIGURE 7:**
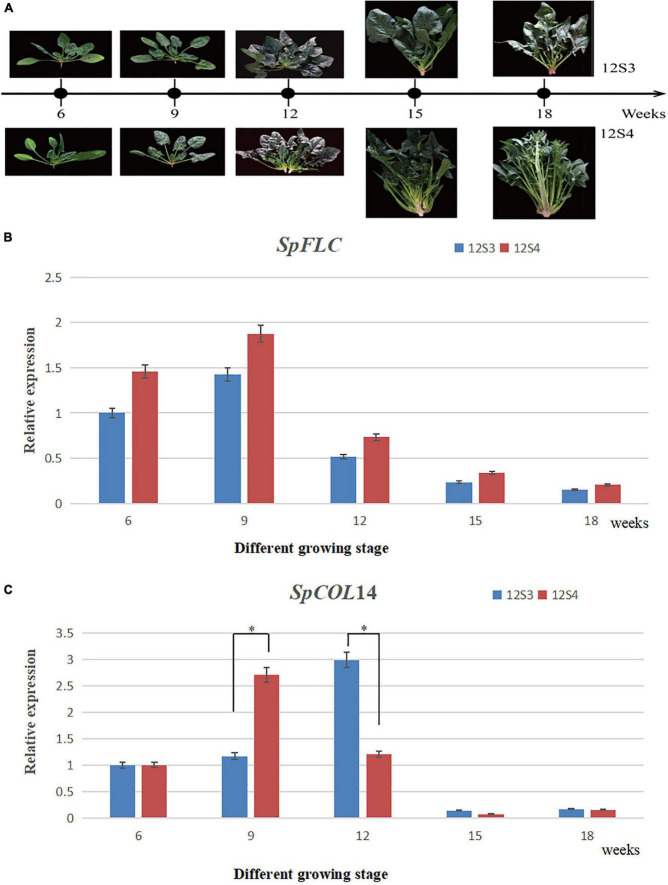
**(A)** Plant materials used in qRT-PCR planted in 2021 spring; **(B)** the expression of *SpFLC*; **(C)** the expression of *SpCOL14*. * Represented significant difference (*P* < 0.05).

Except for *SpFLC* and *SpCOL*14, no other genes were found to be related to bolting and flowering genes, such as *FT*, *SOC*1, and *FLOWERING LOCUS D* (FD) that were reported in other plants (Hideki et al., 2021). Interestingly, in this 0.56 Mb region, we found some transcription factors that regulate the various stages of plant growth and development, including *Spo04911*, which is an NAC domain-containing protein that plays a role in regulating plant growth and stress resistance, and *Spo04943*, which is a MADS-box factor with an *AGAMOUS*-*LIKE* 9 homolog to floral organ development. The factors may play some minor roles in affecting bolting regulation, but the gene regulatory network will be identified in the future with technological progress.

### Expression Analysis of the Candidate Genes Controlling the Spinach Bolting Trait

We assessed the expression patterns using qRT-PCR analysis between 12S3 and 12S4 at different growth stages to further assess the two candidate genes (Table 2). 12S4 bolted after 15 weeks, while the bolting time of 12S3 was after 18 weeks (Figure 7B). Although the expression of these two candidate genes at the five stages were significantly different, a similar change trend of expression levels of *SpFLC* was found both in 12S3 and 12S4, with a high level observed in both at week 9. However, the expression level of *SpCOL*14 between the 12S3 and 12S4 plants was associated with differences in the phenotype; in 12S4 (Figure 7A), the expression of *SpCOL*14 showed a high level at week 9, while 12S3 showed high expression at week 12 (Figure 7C). These results suggested that *SpCOL*14 could potentially be the key candidate gene controlling bolting in spinach.

## Discussion

As a green leafy vegetable, spinach can lose its flavor and thus commodity value in the reproductive stage (Abe et al., 2014). Bolting signifies the first transition between the vegetative and reproductive periods, thus rendering it a criterion of the reproductive stage. This study identified a novel QTL strongly associated with bolting time in BC_1_ and F_2_ populations by KASP and SLAF technology within 2 years.

In an earlier study, the QTLs for bolting in spinach were verified in many groups. Based on a SNP linkage genetic map, three QTLs associated with bolting and flowering (two at P01 and one at P02) were found in Chan-Navarrete et al. (2016), and three SNP markers (AYZV02001321_398, AYZV02041012_1060, and AYZV02118171_95) were screened by Chitwood et al. (2016). With the genome Sp75 sequences, QTLs for bolting were mapped to 44.7 to 50.5 Mb of chromosome 2 (Xu et al., 2017), and SNPs were also discovered on chromosome 2, chromosome 3, and chromosome 5 (Bhattarai et al., 2020). Recently, Hideki et al. (2021) identified three QTLs for bolting time (*q*Bt2.1 on LG2; *q*Bt3.1, and *q*Bt3.2 on LG3) based on the new spinach genome SOL_r1.1. In this study, we fine-mapped a novel QTL *q*BT1.1 for spinach bolting located at 47.56 – 48.12 Mb on chromosome 1, which revealed 45.5% PVE in the two-year average results. The physical location of *q*BT1.1 was close to KMBL29, which had the highest LOD score. The new stable QTL facilitated the confirmation of the major genes responsible for bolting time and allowed for reliable molecular markers for the breeding of bolting resistance in spinach to be explored.

Genes that affect bolting and flowering time have been identified by flower regulatory pathways in *Arabidopsis* (Fornara et al., 2010), which provides a reference for detecting the bolting gene in spinach. Bolting and flowering in spinach are mainly related to the photoperiod pathway. Two *FT* and one *COL* homolog have been isolated in spinach (Abe et al., 2014). Xu et al. (2017) discovered one gene (*Spo00403*) showing high homology to the bolting and flowering gene of *Arabidopsis AGAMOUS-LIKE* 20, and three QTLs (*q*Bt2.1, *q*Bt3.1, and *q*Bt3.2) reported by Hideki et al. (2021) contained *FT*, *FLC*, *AGAMOUS-LIKE* 24 homologs, and *AGAMOUS-LIKE* 22/SVP genes. In the present study, 68 genes were in the major QTL area *q*BT1.1. We detected the target genes using the Flowering Interactive Database^9^ and only found one gene similar to the *Arabidopsis* gene *FLC*, namely the sugar beet *FLC-LIKE* 1 (*BvFL*1). *FLC* is a MADS-box transcription factor that acts as a repressor of floral transition in both the autonomous and vernalization pathways (Sheldon et al., 1999). In sugar beet, which is in the same family as spinach (Amaranthaceae), a notable gene *BvFL*1, which is responsible for bolting in many studies, was shown to act as a repressor of flowering when transformed into an *Arabidopsis FLC* null mutant (Reeves et al., 2007; Mutasa-Göttgens et al., 2010). *FLC* is the key gene in the vernalization requirement as a flowering repressor (Sheldon et al., 2000), and the bolting and flowering of spinach mainly depend on the photoperiod-dependent flowering pathway (Chun et al., 2000a). In our study, the re-sequencing results suggested one SNP variation on *SpFLC*, while the expression of *SpFLC* did not show the same expression levels between 12S3 and 12S4. Given this, *SpFLC* may not be the candidate gene controlling the bolting trait in this study.

Bolting and flowering in spinach are closely related to the photoperiod pathway, The photoperiod pathway gene identified in *Arabidopsis* were not detected in these 68 genes. *SpCOL*14 (*Spo04967*, *CONSTANS LIKE* 14), belongs to the *COL* family and has similar functional domains to *COL14*. The *CO* transcription factor is critical in the photoperiod response and shows characteristic patterns of transcription required for day-length sensing. There are 17 *COL* gene members in *Arabidopsis*, which can be divided into four groups (Group I to Group IV) that have a *CO*, *COL*, and *TOC1* (CCT) domain respectively, mediating the interactions with DNA (Robson et al., 2001). The *CO* family genes have different functions; for example, the expression of *COL*1 and *COL*2 in *Arabidopsis* has no role in bolting and flowering, but delays bolting and flowering in sugar beet (Chia et al., 2008; Ledger et al., 2010), and in soybean, *COL*2 has no significant effect on flowering rhythm, while *COL*5 can promote flowering. However, the functions of individual *COL* genes in *Arabidopsis* have not been fully determined. *COL*14 belongs to Group III of the *COL* family, and comprise one B-box and one CCT domain (Griffiths et al., 2003). With Sanger sequencing, no variations were detected in the coding region of *SpCOL*14 between the early and late flowering parents, while about 900-bp variations were found in the promoters (–730 bp to –1653 bp). According to our qRT-PCR results, the expression of *SpCOL*14 exhibited significant differences between the two parents in different phases, and the expression peak of this gene in the early bolting line appeared several weeks before that of the slow bolting line. In conclusion, *SpCOL*14 is very likely the candidate gene controlling bolting trait in spinach. Further functional analysis of these candidate genes will help elucidate the regulatory mechanism of bolting in spinach. In the further study, we can focus on the different varieties of spinach to take full advantage of the bolting genetic information for breeding.

## Conclusion

In the present study, a major QTL, *q*BT1.1, controlling the bolting trait in spinach, was detected in the BC_1_ and F_2_ populations in two years using KASP and SLAF-seq methods. This QTL was mapped to the same region between 47.56 Mb and 48.12 Mb on spinach chromosome 1 in different segregation populations. This *q*BT1.1 is a novel QTL. In this interval, one gene *Spo04967* (renamed *SpCOL*14) is very likely the candidate gene controlling bolting in spinach. These findings lay a foundation for analysis of the genetic mechanisms underlying spinach bolting and flowering time and can be applied for MAS in spinach breeding.

## Data Availability Statement

The original contributions presented in the study are included in the article/Supplementary Material, further inquiries can be directed to the corresponding author.

## Author Contributions

WQ designed the study. QM and ZL conducted the experiments and analyzed the data. QM wrote the manuscript. WQ, CF, XW, and JW made the revision of the manuscript. ZL, HZ, HS, and ZX prepared and collected the samples. All authors contributed to the article and approved the submitted version.

## Conflict of Interest

CF is employed by Ilera Healthcare LLC. The remaining authors declare that the research was conducted in the absence of any commercial or financial relationships that could be construed as a potential conflict of interest.

## Publisher’s Note

All claims expressed in this article are solely those of the authors and do not necessarily represent those of their affiliated organizations, or those of the publisher, the editors and the reviewers. Any product that may be evaluated in this article, or claim that may be made by its manufacturer, is not guaranteed or endorsed by the publisher.

## Footnotes

1
http://www.spinachbase.org/

2
http://www.primer3plus.com

3
https://www.ebi.ac.uk/Tools/msa/muscle

4
https://www.ncbi.nlm.nih.gov/Structure.cgi

5
http://www.gene-better.cn

6
https://www.transgen.com.cn

7
http://www.takara-bio.com

8
http://spinachbase.org/

9
http://www.phytosystems.ulg.ac.be/florid/
