# Supplementary figures and images for: Quantitative Trait Locus Mapping and Identification of Candidate Genes Controlling Bolting in Spinach (Spinacia oleracea L.)
Source: Front Plant Sci. 2022 Mar 30;13:850810. doi: 10.3389/fpls.2022.850810 (PMC9006512; doi:10.3389/fpls.2022.850810)

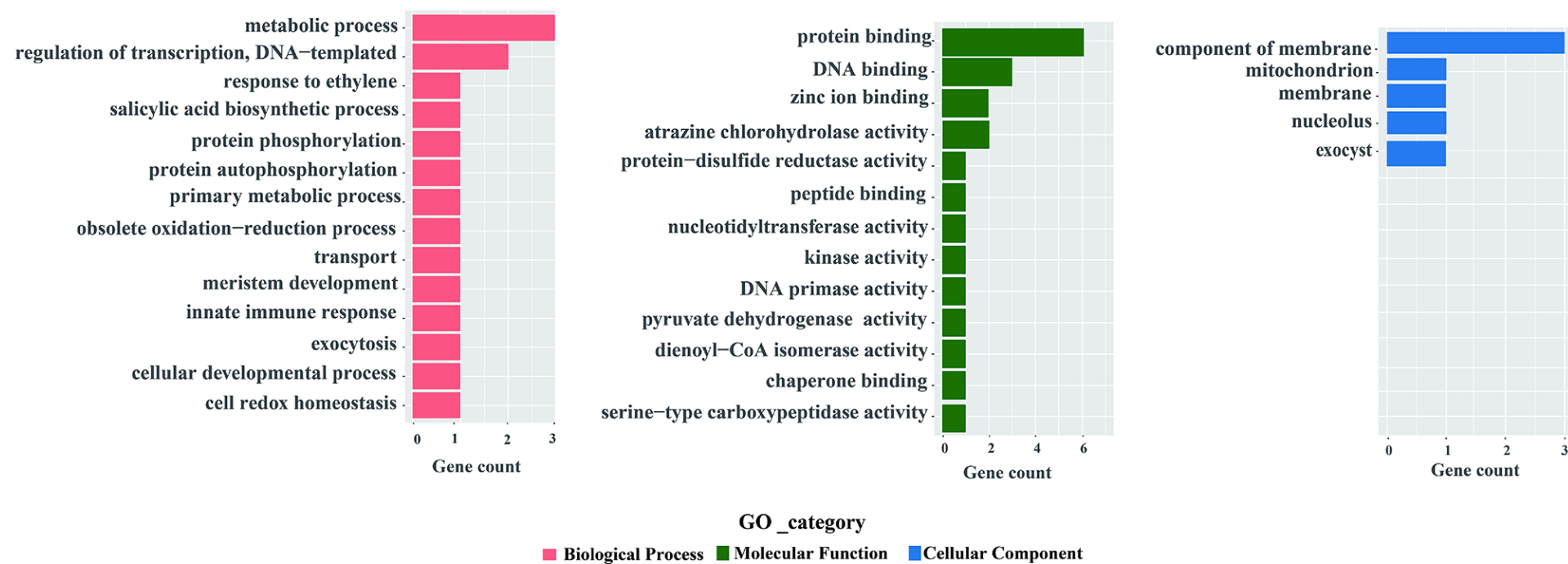

Supplementary Figure 1. The annotation of the 68 genes in the 47.56 - 48.12 Mb on chromosome 1

Supplement: Supplementary file 2 [file Image_1.PDF]
